# Mr. John Wyeth

**Published:** 1907-06

**Authors:** 


Mr. John Wyeth, the head of the firm of John Wyeth and Brother so well known to the medical profession as manufacturing chemists and pharmacists for forty years or more, died at Philadelphia, March 30, 1907. Mr. Wyeth was a gentleman of culture, a scholarly man, genial and companionable, and will be missed by hosts of friends throughout the country.

